# Heart Rate Variability Analysis in Patients with Allergic Rhinitis

**DOI:** 10.1155/2013/947385

**Published:** 2013-02-07

**Authors:** Ming-Ying Lan, Guo-She Lee, An-Suey Shiao, Jen-Hung Ko, Chih-Hung Shu

**Affiliations:** ^1^Department of Otolaryngology, Taipei Veterans General Hospital, No. 201, Section 2, Shipai Road, Beitou District, Taipei 11217, Taiwan; ^2^Institute of Clinical Medicine, National Yang-Ming University, Taipei 112, Taiwan; ^3^School of Medicine, National Yang-Ming University, Taipei 112, Taiwan; ^4^Department of Otolaryngology, Taipei City Hospital, Taipei 103, Taiwan

## Abstract

*Background*. Very few studies investigate the role of the autonomic nervous system in allergic rhinitis. In this study, we evaluated the autonomic nervous system in allergic rhinitis patients using heart rate variability (HRV) analysis. *Methods*. Eleven patients with allergic rhinitis and 13 healthy controls, aged between 19 and 40 years old, were enrolled in the study. Diagnosis of allergic rhinitis was based on clinical history, symptoms, and positive Phadiatop test. Electrocardiographic recordings on the sitting and supine positions were obtained for HRV analysis. *Results*. In the supine position, there were no significant statistical differences in very-low-frequency power (VLF, ≤0.04 Hz), low-frequency power (LF, 0.04–0.15 Hz), high-frequency power (HF, 0.15–0.40 Hz), and the ratio of LF to HF (LF/HF) between the patient and control groups. The mean RR intervals significantly increased, while LF% and LF/HF significantly decreased in the patient group in the sitting position. Moreover, mean RR intervals, LF, and LF/HF, which were significantly different between the two positions in the control group, did not show a significant change with the posture change in the patient group. *Conclusion*. These suggest that patients with allergic rhinitis may have poor sympathetic modulation in the sitting position. Autonomic dysfunction may therefore play a role in the pathophysiology of allergic rhinitis.

## 1. Introduction

Allergic rhinitis is an IgE-mediated inflammation of the nasal mucosa that occurs when an allergen triggers a sensitized immune system. It is considered as a multifactorial disease induced by gene-environment interactions [1]. Various inflammatory cells, cytokines, chemokines, mediators, and adhesion molecules are all involved in the mechanism of allergic inflammation [1]. Recently, neurogenic inflammation has been proposed as an important mechanism [2, 3]. Since nociceptive, parasympathetic, and sympathetic nerves comprise a complex nervous system in the nasal mucosa, they play critical roles in the rapid response to physical and chemical reaction by regulating glandular, vascular, and other processes in defensive and homeostatic functions of the nose [1, 4].

Heart rate variability (HRV), a noninvasive and reliable assessment of both theheartand autonomic nervous system (ANS), measures the degree of fluctuation of the beat-to-beat differences in cardiac rhythm [5]. Either the time-domain method or frequency-domain method in HRV analysis has been used in studies for evaluating the relationship between a specific disease and the autonomic function. However, very few studies have investigated the role of ANS in allergic rhinitis. Ko et al. found that central ANS activities, especially the sympathetic nervous system, significantly correlated with changes to the nasal airway during postural change in normal subjects [6]. Yokusoglu et al. found that HRV indices, which predict parasympathetic predominance, increased in allergic rhinitis patients [7]. The study of Taşcilar et al. implied sympathetic withdrawal and parasympathetic predominance in children with allergic rhinitis and proposed that autonomic imbalance may be involved in the pathophysiology [8]. The study evaluated the autonomic nervous system in adult patients with allergic rhinitis using HRV analysis.

## 2. Materials and Methods

### 2.1. Patients and Controls

Eleven patients (7 males and 4 females) aged between 19 and 39 years diagnosed with allergic rhinitis were enrolled. The diagnosis of allergic rhinitis was based on clinical history, symptoms, and positive Phadiatop test. ThePhadiatoptest, a specific Ig-E test for multiple allergens, had been used as a screening test in allergic rhinitis [9]. Patients with nasal polyposis and sinusitis or a history of asthma, diabetes mellitus, hypertension, stroke, cardiovascular disease, or autoimmune disease were excluded. The patients were not allowed to use any oral or topical nasal medications two weeks before the HRV study. Gender- and age-matched 13 healthy subjects (5 males and 8 females) without a history of allergic rhinitis represented the control group. The study was approved by the institutional review board of the hospital. 

### 2.2. Heart Rate Variability Analysis

Detailed procedures for HRV analysis were as previously described [10]. The subjects were instructed to sit quietly in a chair for 10 minutes for adaptation to the environment, which was air-conditioned with temperature of 25°C. Two electrodes were placed on the forearms of the subject to record the ECG signals for 5 minutes in the sitting and supine positions for another 5-minute recordings. The ECG signals were recorded in digital format using an ECG amplifier and an 8-bit analog-to-digital converter with a sampling rate of 256 Hz. The ECG signals were then processed using a computer algorithm that identified each QRS complex and rejected ventricular premature complex or noise according to their likelihood in a standard QRS template. Each R-R interval was retrieved, resampled, and interpolated at the rate of 7.11 Hz to construct an evenly sampled smooth contour of heartbeat in the time-domain. 

The power spectrogram of heartbeat was acquired using the fast Fourier transform (FFT) of the heartbeat contour. The power spectrum was subsequently quantified into standard frequency-domain measurements as defined in the literature [11], including a very-low-frequency power (VLF, ≤0.04 Hz), low-frequency power (LF, 0.04–0.15 Hz), high frequency power (HF, 0.15–0.40 Hz), and the ratio of LF to HF (LF/HF). The LF, HF, and LF/HF were logarithmically transformed to correct the skewness of distribution. In general, the HF represented the parasympathetic activity of ANS, whereas the LF and LF/HF represented the sympathetic modulation. LF was also presented in percentage (%), which represented the LF component in proportion to the total power. 

### 2.3. Statistics

The Student's *t*-test was used for comparing parametric variables and the Mann-Whitney *U* test was used for comparing nonparametric variables, between the allergic rhinitis group and the control group. Data between the sitting and supine positions in each group were compared using paired sample *t*-test for parametric variables and Wilcoxon's signed-rank test for nonparametric variables. Statistical comparisons were analyzed using the SPSS 12.0 software. A *P* < 0.05 was considered statistically significant. 

## 3. Results

The demographic and clinical characteristics of the patients were summarized in Table 1. The average age of the allergic rhinitis group (7 males and 4 females) was 29.5 years (range, 22–40 years). The average age of the control group (5 males and 8 females) was 29.77 years (range, 19–29 years). There were no statistical differences in age and sex between the two groups (*P* > 0.05). 

In the control group, the RR intervals in the supine position were significantly greater than those in the sitting position, indicating a significant decrease in the rate of heartbeats in the supine position (Table 2). However, this was not found in the allergic rhinitis group. The LF and LF/HF significantly decreased in the supine position in the control group, but did not change after postural change in the allergic rhinitis group (Table 2). This implied that patients with allergic rhinitis might have poor sympathetic modulation in the sitting position than normal controls.

**Figure 1:**
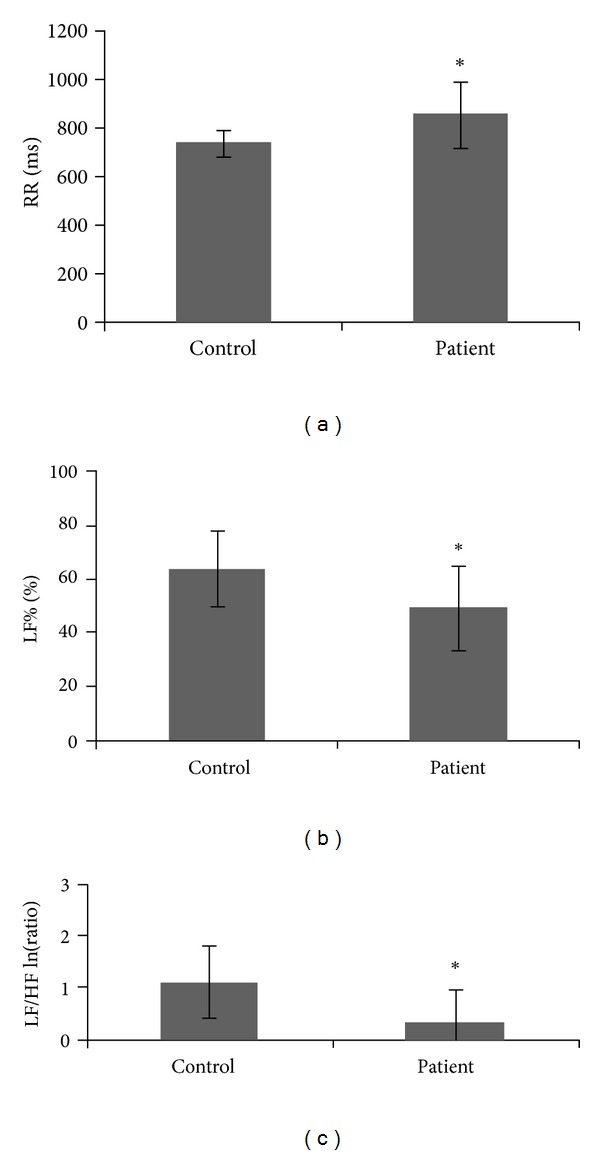
(a) RR interval, (b) LF%, and (c) LF/HF in the sitting position between the control and patient groups. *P < 0.05.

Statistical comparisons of HRV analysis between the two groups in different positions were further analyzed. In the supine position, all variables were not significantly different between the two groups. However, in the sitting position, the RR intervals were significantly higher, while LF% and LF/HF were significantly lower in the allergic rhinitis group than in the control group (Table 2, Figure 1). 

## 4. Discussion

The role of antigen-specific IgE-mediated hypersensitivity reactions in the pathogenesis of allergic rhinitis is well known. However, it seems to be insufficient to account for all of the disease symptoms. It is known that optimal function of the nose depends on a delicate balance of adrenergic, cholinergic, and sensory components of the ANS. In the nasal mucosa, resistance vessels that regulate regional blood flow are composed of arterioles, capillaries, and arteriovenous anastomosis, while capacitance vessels that determine nasal patency are composed of veins and sinusoids [12]. Vessel tone is largely controlled by autonomic sympathetic fibers, while the parasympathetic stimulation largely affects glandular secretions with a little effect on blood flow [12]. An imbalance between the parasympathetic and sympathetic nervous system in sinonasal cavity is thought to play a role in inflammatory nasal and sinus disorders [13]. Many ANS neuropeptides with effects on the nasal mucosa play roles in maintaining homeostasis of the nasal cavity [14]. These are evidences that ANS dysfunction may be a contributory factor in pathogenesis of allergic rhinitis.

Unfortunately, few studies have investigated the ANS function in allergic rhinitis patients using different methods [7, 8, 15]. Ishman et al. used five quantitative ANS tests to evaluate ANS in allergic rhinitis patients and identified that sympathetic hypofunction in all of the allergic rhinitis patients studied [15]. Yokusoglu et al. used only the time-domain method of HRV analysis and found that parasympathetic predominance increased in such patients [7]. When the frequency-domain method of HRV analysis was included to evaluate ANS in children with allergic rhinitis, there were similar results with sympathetic withdrawal and parasympathetic predominance [8]. All of these studies proposed that autonomic imbalance might be involved in the pathophysiology of allergic rhinitis. In the present study, noninvasive and simple HRV analysis was used to measure ANS function. Short-term recordings of the frequency-domain method were more time saving and practical than the traditional 24-hour recordings of the time-domain method used in the Tascilar study and were easier to conduct than the five ANS tests of the Ishman study.

Ko et al. found a decrease in the rate of heartbeats during posture change from sitting to supine [6] and supposed that the decrease was related mainly to the withdrawal of sympathetic activity rather than a change in parasympathetic activities. In the present study, this phenomenon was not found in patients with allergic rhinitis. Significant changes in LF and LF/HF during postural changes in the control group, which represented the overall ANS activities and sympathetic modulation, respectively, were not found in the allergic rhinitis group. Comparing all of the variables in HRV analysis between the two groups in different positions, there was no significant difference in the supine position. However, the RR interval, LF%, and LF/HF differed significantly in the sitting position. The LF% and LF/HF were significantly lower in the allergic rhinitis group than in the control group, indicating that allergic rhinitis patients have poor sympathetic modulation in the sitting position than normal subjects. These results further support the study by Ishman et al., wherein sympathetic hypofunction is specifically noted in patients with allergic rhinitis [15].

There may be some inconsistent results among the HRV variables in the present study compared to other studies, perhaps due to different ethnic populations, different ages, various sample sizes, and different ANS methods used. However, all of these studies, as well as the current study, identify autonomic dysfunction in patients with allergic rhinitis. Further studies with a larger series of patients and advanced basic research on molecular mechanism of ANS abnormalities will help to elucidate the pathogenesis of allergic rhinitis.

## 5. Conclusion

Patients with allergic rhinitis seem to have poor sympathetic modulation in the sitting position than the control subjects. This preliminary study suggests that autonomic dysfunction may play a role in the pathophysiology of allergic rhinitis.

## Figures and Tables

**Table 1:** Demographic and clinical characteristics of patients.

Characteristics	Patients (*n* = 11)
Age, mean (yrs)	29.5
Gender	
Male	7 (63.6%)
Female	4 (36.4%)
Symptoms	
Sneezing	9 (81.8%)
Rhinorrhea	9 (81.8%)
Nasal obstruction	11 (100%)
Nose itching	8 (72.7%)
Eye itching	7 (63.6%)
Symptom duration (months)	109.9
Positive Phadiatop test	11 (100%)
Classification	
Intermittent AR	1 (9.1%)
Persistent AR	10 (90.9%)

AR: allergic rhinitis.

**Table 2:** Statistical comparison of HRV variables in control group and patient group.

	Control		Patient		*P* value			
	Sitting (mean ± SD)	Supine (mean ± SD)	Sitting (mean ± SD)	Supine (mean ± SD)	Control (sitting versus supine)	Patient (sitting versus supine)	Sitting (control versus patient)	Supine (control versus patient)
RR interval	735.3 ± 58.3	815.8 ± 78.0	855.7 ± 135.9	892.18 ± 155	**<0.001**	0.656	**0.017**	0.132
VLF	6.5 ± 0.9	6.5 ± 1.2	6.8 ± 0.7	6.87 ± 1.5	0.972	0.858	0.421	0.535
LF	6.4 ± 0.8	5.9 ± 1.2	6.2 ± 1.0	5.95 ± 1.1	**0.023**	0.523	0.546	0.902
HF	5.3 ± 1.0	5.3 ± 0.8	5.9 ± 1.1	5.85 ± 1.2	0.499	0.886	0.207	0.147
TP	7.5 ± 0.7	7.3 ± 1.0	7.6 ± 0.8	7.66 ± 1.2	0.468	0.927	0.655	0.454
LF%	63.8 ± 14.1	54.5 ± 18.6	49.2 ± 15.5	43.45 ± 14.6	0.056	0.354	**0.025**	0.125
LF/HF	1.1 ± 0.7	0.7 ± 0.8	0.3 ± 0.7	0.10 ± 0.7	**0.044**	0.374	**0.019**	0.091

VLF: very-low-frequency power; LF: low-frequency power; HF: high-frequency power; TP: total power; LF%: LF percentage; LF/HF: ratio of LF to HF.
